# Reusable design: A proposed approach to Public Health Informatics system design

**DOI:** 10.1186/1471-2458-11-116

**Published:** 2011-02-18

**Authors:** Blaine Reeder, Rebecca A Hills, George Demiris, Debra Revere, Jamie Pina

**Affiliations:** 1 Biobehavioral Nursing and Health Systems, School of Nursing, Box 359442, University of Washington, Seattle, WA 98195, USA; 2 Medical Education and Biomedical Informatics, School of Medicine, Box 357240, University of Washington, Seattle, WA 98195, USA; 3 Department of Health Services, School of Public Health, Box 354943, University of Washington, Seattle, WA 98195, USA

## Abstract

**Background:**

Since it was first defined in 1995, Public Health Informatics (PHI) has become a recognized discipline, with a research agenda, defined domain-specific competencies and a specialized corpus of technical knowledge. Information systems form a cornerstone of PHI research and implementation, representing significant progress for the nascent field. However, PHI does not advocate or incorporate standard, domain-appropriate design methods for implementing public health information systems. Reusable design is generalized design advice that can be reused in a range of similar contexts. We propose that PHI create and reuse information design knowledge by taking a systems approach that incorporates design methods from the disciplines of Human-Computer Interaction, Interaction Design and other related disciplines.

**Discussion:**

Although PHI operates in a domain with unique characteristics, many design problems in public health correspond to classic design problems, suggesting that existing design methods and solution approaches are applicable to the design of public health information systems. Among the numerous methodological frameworks used in other disciplines, we identify scenario-based design and participatory design as two widely-employed methodologies that are appropriate for adoption as PHI standards. We make the case that these methods show promise to create reusable design knowledge in PHI.

**Summary:**

We propose the formalization of a set of standard design methods within PHI that can be used to pursue a strategy of design knowledge creation and reuse for cost-effective, interoperable public health information systems. We suggest that all public health informaticians should be able to use these design methods and the methods should be incorporated into PHI training.

## Background

To understand the need for adoption of standard Public Health Informatics (PHI) design methods requires a brief background of PHI, the environment in which public health information systems are used and the unique challenges encountered in designing information systems for use in public health practice.

### The unique field of Public Health Informatics: population-focused and diverse

Public Health Informatics is the "systematic application of information and computer science and technology to public health practice, research, and learning that integrates public health and information technology"[1]. And, as stated by Yasnoff et al.[2], PHI needs to use a "systematic and informed approach to the application of information science and technology in order to take full advantage of its potential to enhance and facilitate public health activities." Such activities include:

• Promotion of the health of populations as "opposed to the health of specific individuals."

• Prevention of disease and injury "by altering the conditions or the environment that put populations of individuals at risk."

• Prevention at all vulnerable points in the "causal chains leading to disease, injury, or disability ... not restricted to particular social, behavioral, or environmental contexts."

• Reflection of the "governmental context in which public health is practiced "[2].

In the application of information science and technology to population health, the challenges of PHI are unique. For example, unlike clinical informatics, PHI design activities cannot simply focus on an individual's data in an Electronic Health Record (EHR), the transmission of a specific patient visit in a data stream between a health care provider organization and public health agency or an intervention targeted to an individual patient. Instead, PHI design activities must take a more systemic approach that allows access to population-level data with functionality tailored to a variety of uses by different groups of practitioners. Instead of individual patient data, public health information systems need to include and manage entire datasets of patient encounters. Supporting prevention-centered activities moves the focus further up the causal chain of health and is less likely to manifest in an individual patient visit, but might instead be seen as a population-level prevention intervention, e.g., a city-wide anti-tobacco campaign. These prevention activities take place in diverse environments since the interventions may be social, behavioral, environmental or individual interventions. In addition, public health informaticians must always consider the governmental context in which these population-level prevention activities take place. Understanding the health services structure and how that structure affects the work done by public health practitioners is an important step in creating systems that will function within that context.

Diversity in public health practice presents challenges for public health informaticians. A perceived lack of standard work practices across local health jurisdictions on the part of practitioners and administrators complicates public health information system design activities. If each state or local jurisdiction has unique information needs and business processes, then each must make individual design decisions to ensure that their needs are met. Recent efforts have been undertaken to create standards for business processes in the nation's local health jurisdictions. These efforts have uncovered unexpected commonalities[3] as well as developed a taxonomy of common public health work tasks, knowledge and resources[4]. Yet it has been argued that there is a great deal of variability in local health jurisdictions[5] and further research is necessary to document the needs and tasks of public health practitioners within specific local health jurisdictions to design technology to support these needs and tasks[6].

Adding to this diversity of practice and settings is the diversity of national and international settings in which these activities take place. The globalization of public health necessitates a globalization of PHI to address the challenges of disparate data, tools and services within and across nations--both resource-rich and -constrained. Issues of conflicting data standards, the need for interoperable tools for exchanging and sharing data and the need for innovative solutions to address integrated disease surveillance, among many other issues, are driving forces to formalize design strategies in PHI.

The development of public health information systems requires an understanding of the principles, practices, structures and settings in which these systems operate. Although this combination of factors presents unique challenges, PHI is at a point in its evolution as a discipline that public health informaticians can learn from the design theories and experiences of other fields to address these challenges.

### Evolution of public health information systems in the US and Europe: three waves

According to Lumpkin, federal-state public health information systems in the United States have been developed in three "waves"[7]. The first wave began with the independent development of state and federal systems which could not exchange data; the second wave, supported by federal funding to develop state level systems capable of exchanging data using standards in building independent--and therefore costly--state systems[7]; and the third wave which focused on reducing costs through the integration of "the benefits of state-level system development with the tools of software reuse" and a requirement that "each system that is developed must be standards based"[7].

Similarly, in Europe, public health information systems developed in three comparable waves[8]: a first generation of systems focused on collecting basic data on a population and/or the health of a population (for example, birth and death data); a second generation of systems that combined stratifications of time-series data and international comparative data as seen in comparisons of age-standardized breast cancer incidence rates by sex over time and country; and the third generation in the present, with the public health information system that is made possible by information technology advances and is referred to as an ''integrated knowledge system'' in its integration of data, descriptive and analytical information, and evidence-based knowledge[8].

In both the European and US contexts, this current third wave of public health information systems utilizes electronic data exchanges and standards to solve the information needs of public health workers at different organizational levels, across organizations and with various health care and government stakeholders--thus addressing the challenges of population focus and diversity discussed above. While a common approach to this third wave presumes that system designers both have sufficient knowledge of the work of public health practitioners and can systematically integrate this knowledge into system design, past technology failures suggest that this is not the case and numerous studies have underscored the fact that a system will not be used by health professionals in everyday practice unless the system is usable. While a literature review on challenges to adopting or deploying health information systems is beyond the scope of this paper, these challenges are pertinent to the topic of design and PHI. The reader is referred to the work of Kushniruk et al.[9] and Peute et al.[10] for additional information.

### Complex problems and design challenges in public health

Ignoring the user experience has led to a literature replete with numerous examples of health information systems being "turned off" or rejected by health professionals because these systems were developed without an understanding of the information needs, workflow, or architecture needs of system users[11,12]. As previously noted, public health processes and practices can be difficult to define, developing clear descriptions of this work requires concerted effort[13], and a public health information system that meets the needs of one group may simultaneously create more work for other groups of public health practitioners[14].

The focus of public health agencies on the development and implementation of information systems is a relatively new phenomenon. Public health informaticians face significant challenges in designing and implementing flexible, interoperable, usable systems to meet the needs of public health practice. Recommendations for a national agenda for PHI in the United States were not formally outlined until 2001[15] and competencies for public health informaticians that include "expertise in both public health programs and information systems to help design, implement, and manage computer applications that support public health goals"[16] were only finalized in 2009. The outcome is that many public health information systems often came into existence through an ad hoc and informal design and development process[17,18] yet the public health domain represents a complex design setting.

Complex design problems of the type encountered in the public health domain are not new. Following World War II, Weaver defined problems of disorganized complexity and organized complexity[19]. Weinberg discusses problems of disorganized complexity as large, random populations that are subject to statistical treatment while individual problem cases are members of small, structured populations that are subject to individual analysis[20]. Information design in PHI addresses a problem area of organized complexity that is subject to neither individual nor statistical analysis.

Rittel and Webber frame their design discussion by enumerating a list of problems and claim that defining what systems do and planning what they should do in terms of desired outcomes can be difficult, if not impossible in large, societal systems[21]. Buchanan holds that "the problem for designers is to conceive and plan what does not yet exist and this occurs in the context of the indeterminancy of wicked problems" and this "indeterminancy implies there are no definitive conditions of limits to design problems"[22]. Nevertheless, public health practice hinges on information that is managed by information systems and those systems *must *be designed. Our proposed approach for PHI is not to specify design solutions in advance but to specify a flexible process that involves public health practitioners to design solutions for public health systems of organized complexity. This approach is supported by the writings of Cross who summarizes forty years of design and notes that the "wicked problems" characterized by Rittel are more appropriately satisfied by "an 'argumentative', participatory process in which designers are partners with the problem 'owners'" rather than by a rigid, step-wise process[23].

Problem representation in the design of complex systems requires reflection. Simon explicated the formidable task of representing new problems that do not fit with previously known patterns[24] while Lawson reflects on the need for a process that allows problems and solutions to emerge simultaneously and reflect each other[25]. Numerous design thinkers cite the contribution of Donald Schön's model of the "reflective practitioner" and a model of inquiry that relies on interactive problem framing [23,25-27]. In formalizing his approach to scenario-based design, John M. Carroll has built on Schön's metaphor of design as a conversation[28].

We propose that PHI can benefit from design theories and methods developed in other disciplines to improve public health information systems by addressing the unique work of public health practitioners. In the next section we describe six general design problems as defined by Carroll[29] and provide examples of how those problems express themselves in the unique context of the public health domain. We then present a strategy for creating reusable design knowledge using established participatory and scenario-based design methodologies. Finally, we address some potential challenges to the approach we propose.

## Discussion

The overarching challenges associated with system design within the public health context can be described, in part, as traditional design problems. As mentioned previously, Carroll uses a "design as conversation" metaphor for interactive problem-framing and -solving. Carroll and his collaborators, long-time proponents of design methods in Human-Computer Interaction, formalized this approach in scenario-based design and described six general problems in design work[29]. While the philosophy and practice of modern design have a well-documented history[22-24,26,27,30-32] that dates back to John Dewey in the early part of the last century[33,34], we believe that Carroll's approach is a simple way to highlight the main categories of design challenges for public health information systems. To demonstrate how traditional design problems play out in the public health context, in Table 1 we have mapped Carroll's six design problems to public health information examples.

**Table 1:** Design problems and solutions in the public health context

Design Problem	Example Design Problem in the Public Health Context	Reusable Design Solution
**Incomplete Problem Description**		
*Problems in system design rarely make their conditions clear early in the design process*	Few formal needs assessment studies[35]	Needs assessments conducted in coordination with participatory design to clarify problems by learning from their users, users' goals and users' data needs[36]
**Unclear Design Pathway**		
*While there are many possible steps that a designer can take while clarifying a problem description, the best path is not obvious*	Need to understand the work practices of public health practitioners[36]	Participatory design[37] to create design specifications so a Public Health Informatics solution is incorporated into an optimized workflow and environment
**Impact of Design Solutions are Hard to Predict**		
*Although the general type of problem may be understood, the solution to the problem can exceed the problem itself*	Public health systems are designed and deployed without complete knowledge of the working environment system[38]	Create a representation of an intentional future[12] by using Human-Computer Interaction, Interaction Design, User-Centered Design and Contextual Design to understand the public health context.
**Trade-Offs Due to Competition Among Resources**		
*Project components compete for resources, some elements of the project constrain the design of other elements, and these competing interests challenge resolving conflicts between the design elements*	Limited resources mean not all system requirements can be met	Documented design knowledge that allows for comparisons between competing solutions
	Balancing competing characteristics such as sensitivity and false negatives[39,40]	
**Integrated and Interdependent Needs**		
*Collaboration is needed between designers and users to pool knowledge*	Diverse work and diverse information systems[14]	Participatory design that includes a mixture of representative public health work roles and teams
		Create scenarios that include a mixture of representative public health work roles and teams
**Unintended Consequences**		
*Unintended consequences can have a significant impact on users and those outside the originally conceived group of stakeholders*	Data produced by public health systems is used for different purposes by a diverse set of individuals	Use scenario-based design to describe the tasks and activities necessary to deliver essential services
	Health practitioners have complex roles and workflows[41,42]	

Patel and Kaufman frame informatics as a "local science of design"[43], and make the case for the incorporation of design methods from other disciplines to solve practical problems in informatics and set the stage for development of specific guidelines for design within informatics specialties. Although they caution against generalizing widely in the abstract, we believe, as they do, that principles of usability and design can be incorporated within specific domains once a domain has been defined. The strategy we propose is in concert with the development of PHI as a maturing domain in which to incorporate design methods from other disciplines. Specifically, we propose participatory design and scenario-based design as key components in a reusable design strategy. In the following section, we propose a unified strategy for public health information system design based on methods that have been developed in other disciplines.

### Participatory design in health care settings

Participatory design aims to actively engage all stakeholders in the design process to ensure that the system meets their needs and expectations, and ultimately is adopted by the target practitioners. This approach represents a departure from traditional approaches to information system design in that it promotes rapid prototyping and iterative approaches to implementation rather than segregated phases of design and technical production. The concept of participatory design originated in the Scandinavian countries where research projects on user participation and involvement date back to the early 1970s [44]. These early projects focused primarily on empowering workers in actively participating in the design and implementation of systems and workflow processes to improve working conditions. Some of the early work also focused specifically on health care. The Florence project[45] followed a participatory design approach to empower nurses to play an active role in the development of work processes and information technology applications in hospitals.

The participatory design approach began a slow adoption process in the US in the late 80s and early 90s, however it has not been extensively employed in health care settings[46]. Case studies and small scale efforts demonstrate its potential; Sainfort et al.[47] point out how user involvement can ensure four specific qualities for information system interfaces that are appropriate for clinical providers and patients, namely multimodal, personalized, context aware and adaptive. Involving practitioners in the early design stages can maximize these qualities as a system prototype is developed and ultimately implemented. As Pilemalm and Timpka point out, participatory design methods in the disciplines of health informatics have been mainly applied to the development of small-scale systems with homogeneous user groups in local settings[48]. They present a participatory design framework for large-scale system design. The proposed framework was designed and validated within a PHI project aimed at developing a system for 175,000 users.

### Scenario-based design

Scenario-based design is a methodology that places the focus of system design on the activities of the people who use an information system rather than the system itself or the capabilities of technology[28,49,50]. It is a participatory method in that it solicits the needs and values of work practitioners by bringing them directly into the design process. Scenarios are stories that use everyday language to describe people and their work activities[28]. These narratives can be used to communicate with laypersons who may lack technology or software design training[51]. The use of everyday language to describe narratives of use makes scenario-based design a practical methodology when applied in a variety of contexts, including that of public health.

### Reusable design knowledge in the public health context

Reusable design knowledge is design advice that is "generalized so it can be reused in a wide range of contexts"[52]. Reusable design knowledge can come in the form of data standards for information storage and exchange[40,53-57], software design patterns used by programmers when they develop a system[58-63] or installable software that is implemented in multiple settings[64]. However, this discussion pertains to design methods and the designed activities and workflows that result from those methods.

Whittaker et al. propose the notion of shared tasks called "reference tasks" for use by designers in different application domains and state "[t]he goal of reference tasks is to capture and share knowledge and focus attention on common problems"[65]. Scenario-based design is one means to accomplish the goal of creating reference tasks in the public health domain as Carroll asserts that "[s]cenarios can also be abstracted and categorized, helping designers to recognize, capture and reuse generalizations"[28]. In addition, Wahid has proposed the reuse of positive and negative claims about particular design moves used to weigh trade-offs in scenario-based design[66] while Sutcliffe has done considerable work to advance the effective reuse of scenarios[52,67,68]. Within the context of public health we propose using scenario-based design to describe the tasks and activities necessary to deliver public health activities and to "jointly identify reference tasks, collect data, analyze the tasks, and disseminate and make use of the results"[65]. We believe that these processes and the resulting products will facilitate the reuse of design knowledge and resource savings in the public health domain.

While there is growing work in identifying and documenting information needs for public health practitioners[69], we propose that the next steps in formalizing this documentation and transforming it into reusable design knowledge should include the development of scenarios of use to inform the design of information systems. Collaboration and practitioner involvement are two important aspects of developing useful scenarios. Our experiences have shown that public health practitioners are enthusiastic participants in the scenario development process[14,70]. Scenarios can be developed in a participatory fashion using data collected from interviews, focus groups, surveys and the review of artifacts such as paper documents and electronic files.

The dissemination of reusable design knowledge is important if the goal of reducing work across public health jurisdictions is to be realized. Therefore a plan for taking advantage of existing public health groups and communities of practice should be considered as part of a formal dissemination strategy. By making scenarios available through existing information dissemination channels, public health informaticians and system designers could pick and choose scenarios tailored to the work practices of particular roles and the size of a local health jurisdiction.

Scenario-based design has the potential to offer great improvement in the information systems developed for public health. Aside from encouraging the development of more useful systems, scenario-based design also offers savings in time and resources. Reusing design knowledge can make systems less expensive to develop and offers a potential reduction in failed and resource intensive one-off development projects.

### Potential challenges to the approach

Regardless of size, available resources, or geographic location, each public health organization addresses the most pressing population health concerns of its community--which may explain the variety of public health information systems[69]. As previously discussed, public health organizations also maintain unique work practices that fulfill the requirements of their policy makers, and honor the organizational culture of the individual group. Before widespread Internet access provided a mechanism for public health agencies to exchange and report information electronically, efforts to standardize information systems across local health jurisdictions were limited. Now, as the argument for electronic information exchange becomes more compelling, there is a need to reevaluate the design of information systems in public health.

The approach to information system design within different communities of practice has, so far, reflected the disparate organization of local and state public health agencies. With few means to acquire information systems that have been validated for interoperability, and facing an obligation to optimally address public health concerns despite this gap, public health organizations have found information management solutions through independent development, or through the purchase of commercially available systems. In either case, information management systems are designed to interact with a very limited scope of external entities, thereby reducing the value of important public health data. Without a concerted effort to integrated formal design principles into public health, this trend will continue.

While this paper has focused largely on Public Health Informatics efforts in the United States, it addresses concerns of system design that are relevant to international public health efforts. Public Health Informatics efforts must be informed by the governmental context in which they operate and the environment that obtains in the United States is but one example in the set of all international examples. Working directly with each community of practice, and building a knowledgebase of activities in each, is an essential component of the reusable design approach we propose, regardless of public health environment. For example, in developing countries where resources and capacity are limited, there are several initiatives using participatory design methods to understand the information needs of users as well as the scalability and sustainability of national information systems for providing access to information and supporting the collection, handling and dissemination of health data[71,72].

## Summary

While the technical capacity to develop robust information systems has existed for many years, we have yet to identify the consummate model of public health information processes and develop systems to support those processes. Public Health Informatics can play a role in helping to resolve public health policy challenges, and in the process, define the necessary systems to promote population health, improve PHI as a discipline, and advance all the disciplines of biomedical and health informatics.

The major public health challenges in the coming decade will be solved with the help of policy changes. Information systems are technology artifacts that implement the processes defined by policy. We believe that the policies described in the "National Agenda for Public Health Informatics"[15] should be supported with even more specific policies related to design methods. Additionally, we assert that a policy failing to describe well-tested processes will result in an information system that fails to make optimal use of the information that public health practitioners carefully collect and analyze. Policy should promote the use (and reuse) of design for the development of information systems grounded in public health practice.

Policy changes in the Unites States are already transforming the health IT landscape. The information designs that result in response to these changes will contribute to the global health informatics conversation. Financial incentives for meaningful use of EHR technology reinforce the importance of health information exchange between providers and public health organizations. As policy changes place a greater emphasis on the design and use of effective health IT, the need for systematic design approaches should increase. In the case of compliance with meaningful use criteria, methods for design reuse can facilitate work that has already begun for the exchange of health information within and between provider and public health systems[73]. The resulting design knowledge should include specification of how, and with whom, systems interoperate.

As we have suggested throughout this paper, identifying the information needs of public health practitioners is a foundational step toward developing a design strategy for PHI. There are many examples of information system failure since the advent of computing systems in the workplace when target audiences are excluded from the design process. Scenario-based design is a proven methodology for information system design but has not been applied widely within the public health domain. When coupled with methods of design-based inquiry and practitioner participation, it can be a powerful tool to move quickly and efficiently from needs and task documentation to prototypes to implemented systems.

Working directly with each community of practice and building a knowledgebase of activities in each is essential. By including practitioners in a reusable design strategy for public health, we aim to increase the likelihood of future information system adoption in public health practice and, by extension, increase the likelihood of quality public health service delivery. We suggest, as part of the PHI reusable design effort, that information needs studies and technology use cases should be regularly documented and indexed for ready access by informaticians and others. Future work toward a reusable design strategy will include a survey of public health practitioners about their willingness and the ways they would like to participate in design work and a systematic review of design methods used within the specialized corpus of public health technology knowledge.

We believe that the identification and consistent use of rigorous methods for system design will help move the discipline of Public Health Informatics forward. Toward this end, we believe that the design methods outlined in this paper fit into the applied, empirical and theoretical areas that should be incorporated into PHI practice and curricula for professional training at the undergraduate and graduate levels.

## Competing interests

The authors declare that they have no competing interests.

## Authors' contributions

BR conceived the idea for the manuscript, participated in coordination of research and writing of all manuscript revisions. RH participated in coordination of research and writing of all manuscript revisions. GD contributed material on participatory design and participated in writing of all manuscript revisions. DR contributed material on socio-technical and international issues and participated in writing of final manuscript revisions. JP participated in coordination of research and writing of manuscript revisions. All authors read and approved the final manuscript.

## Pre-publication history

The pre-publication history for this paper can be accessed here:

http://www.biomedcentral.com/1471-2458/11/116/prepub
